# What is known about digital literacy, digital inclusion and attitudes to digital health tools among older adults undergoing surgery? A systematic review and narrative synthesis

**DOI:** 10.1093/ageing/afag165

**Published:** 2026-06-08

**Authors:** Amelia M Paveley, Harriet M Hall, Judith S L Partridge, Jugdeep K Dhesi

**Affiliations:** Ageing and Health Department, Guy’s and St Thomas’ NHS Foundation Trust, London, England, UK; Ageing and Health Department, Guy’s and St Thomas’ NHS Foundation Trust, London, England, UK; Ageing and Health Department, Guy’s and St Thomas’ NHS Foundation Trust, London, England, UK; School of Life Course and Population Sciences, Faculty of Life Sciences and Medicine, King’s College London, London, UK; Ageing and Health Department, Guy’s and St Thomas’ NHS Foundation Trust, London, England, UK; School of Life Course and Population Sciences, Faculty of Life Sciences and Medicine, King’s College London, London, UK

**Keywords:** perioperative, digital health literacy, digital access, health inequality, ageing, older people, systematic review

## Abstract

**Background:**

Older adults, who are often living with frailty, multimorbidity and cognitive impairment, frequently present to perioperative services. Digital perioperative tools can be used to improve patient experience and outcomes. Little is known about digital literacy, inclusion and attitudes to digital tools, and barriers and facilitators to usage, in the perioperative setting for older surgical patients.

**Methods:**

Medline, Embase, CINAHL and Cochrane Library were searched from inception to February 2025. PRISMA guidelines were utilised and the study was registered on PROSPERO. Heterogeneity of study methods and reported outcomes precluded meta-analysis. Gough’s Weight of Evidence Framework was used to assess quality of evidence.

**Results:**

Twenty-four studies were included for narrative synthesis. No studies used a validated measure of digital literacy. Digital access was variably reported. Older adults were less likely than younger people to use the internet, wearable technology and telemedicine. Many studies excluded individuals without access to their own devices, limiting generalisability. Older adults expressed positivity about digital tools but did not universally prefer digital platforms. Barriers to engagement included digital exclusion, poor device usability and patient factors. Support through training or caregivers facilitated engagement.

**Conclusion:**

Digital perioperative tools are increasingly available but there is a paucity of data on digital literacy, access and usage in the older surgical population. Studies frequently excluded those living with frailty, multimorbidity and cognitive or sensory impairments. Further research is needed to understand the needs, attitudes and digital health literacy of this population, to facilitate codesign of accessible, acceptable and effective perioperative digital health tools.

## Key points

- Many perioperative digital innovations are emerging, but few have been robustly studied in older adults having surgery.
- Studies frequently excluded those with cognitive, sensory or functional impairment, frailty and without digital access.
- No studies used a validated measure of digital literacy or digital health literacy.
- Key barriers to digital use include limited digital access, high cost and reliance on support to use digital tools.
- Codesign with older adults is essential to design accessible, implementable perioperative digital interventions.

## Introduction

The global population is ageing [1]. With surgery offering definitive management for many age-related diseases, an increase in older adults undergoing surgery is observed. In England, one in five over 75s is predicted to have surgery annually by 2030 [2]. Older adults are more likely to be living with frailty, multimorbidity, polypharmacy and physical or cognitive impairment placing them at increased risk of adverse postoperative outcomes.

The perioperative pathway encompasses the whole surgical journey from preoperative referral and assessment through to intraoperative events and postoperative recovery. There is a growing interest in perioperative digital tools—such as wearables, mobile applications and telemonitoring—particularly in the context of prehabilitation and rehabilitation [3]. Their development must consider health and eHealth literacy, digital inclusion and patient acceptability.

The World Health Organisation defines health literacy as the ability to engage with health and information services. Personal skills and social resources are required to access, understand and use information to make health decisions [4]. NHS England describes digital literacy as the capabilities needed to live, work, participate and thrive in a digital society [5]. ‘Digital health literacy’ or ‘eHealth literacy’ combines these concepts. Essential digital skills include basic device operation, connecting to Wi-Fi, using browsers and applications and keeping login information secure [6]. This combination of adequate digital skills and access constitutes ‘digital inclusion’ [7]. Common tools to measure digital literacy include the eHealth Literacy Scale (eHEALS), the eHealth Literacy Questionnaire (eHLQ) and the Digital Health Literacy Instrument (DHLI) [8, 9].

In 2021, two fifths of adults over 75 years in England were digitally excluded through a lack of digital skills and access to devices [10]. In the UK, 4% of adults lack internet access, rising to over 30% of adults over 65 years and up to 60% of older adults with physical, sensory or cognitive impairment [11–13]. Younger age, higher education and income levels, more internet use and better social support are associated with better eHealth literacy [14, 15]. One study estimated a quarter of surgical inpatients have inadequate digital health literacy or ability to navigate digital systems [16].

As older people form an increasing proportion of patients undergoing surgery, digital innovations must be codesigned with this heterogenous group. Perioperative eHealth interventions for older surgical patients appear feasible but better understanding of health and eHealth literacy, and digital inclusion is required to inform future innovation [17].

This review aims to answer, for older adults having surgery:

1. What is known about the level of digital literacy or digital health literacy, and how is this measured?
2. What digital access and resources are available to this population?
3. What are the attitudes of older adults towards digital perioperative intervention?
4. What barriers and facilitators influence engagement, use and uptake of digital interventions in the perioperative period?

## Methods

This systematic review was carried out according to the Preferred Reporting Items for Systematic Reviews and Meta Analyses (PRISMA) statement protocol and registered in the International Prospective Register of Systematic Reviews (PROSPERO ID: CRD42022345647) [18]. The PRISMA checklist can be found in Appendix S1 of the Supplementary Data section.

### Search strategy and selection criteria

Four databases were searched from inception to 13 February 2025: Medline (PubMed), Embase (Ovid), CINAHL and Cochrane Library. The search used variations of ‘health literacy,’ ‘eHealth’ and ‘geriatric’ (Appendix S2). Screening was managed using a web-based systematic review software (Rayaan), with titles and abstracts screened by two reviewers (AP, HH) and full texts reviewed independently. Discrepancies were resolved by discussion with senior authors (JP, JD).

Included studies evaluated in older adults undergoing surgery:

- Digital literacy or digital health literacy.
- Availability of internet, electronic devices or other digital tools.
- Attitudes towards digital intervention in perioperative care.
- Barriers and facilitators to using digital interventions in perioperative care.

**Figure 1:**
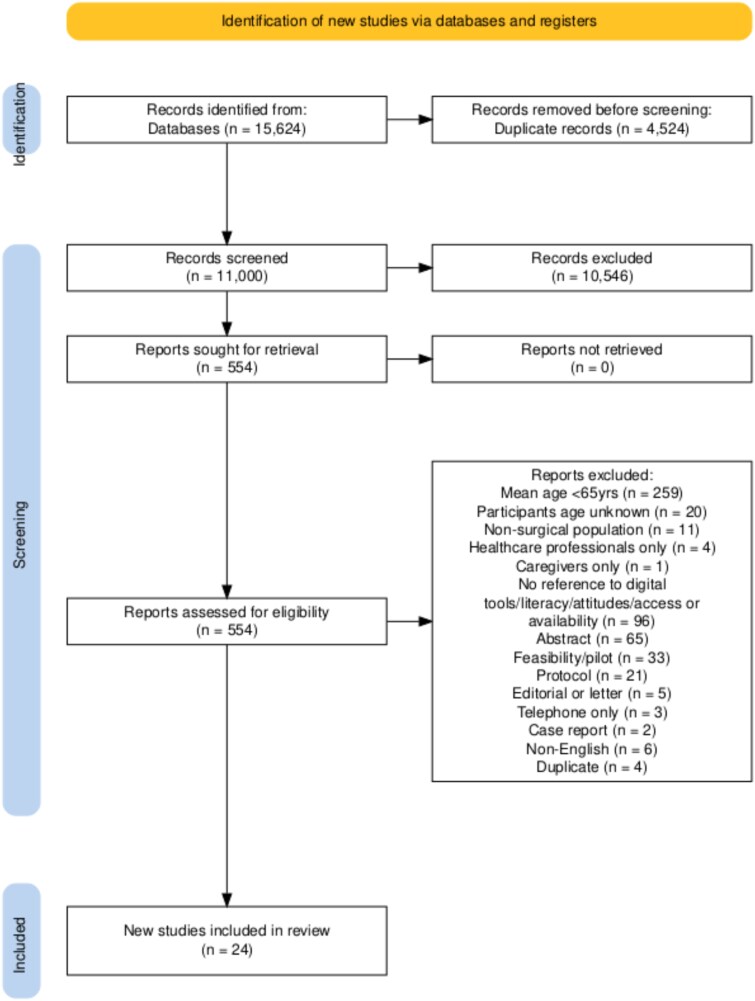
PRISMA diagram for systematic reviews.

A mean study population age of ≥65 years was required. Experimental, quasi-experimental and observational studies were considered. Exclusions were reviews, case reports, editorials, feasibility studies, abstracts, protocols and studies of digital tools without reporting digital or digital health literacy, attitudes or barriers/facilitators. Studies looking at health literacy without a digital component, telephone-only interventions and non-English studies were also excluded. Full exclusion criteria are shown in the PICO diagram (Appendix S3), and reasons for exclusion at full text review are shown in the PRISMA diagram (Figure 1).

### Data extraction and analysis

Data extraction included study characteristics (design, sample size, surgical specialty), patient demographics (age, education, socioeconomic status, functional level, frailty), digital tools used, measures of digital literacy and health literacy, and qualitative and quantitative assessments of attitudes, barriers and facilitators.

A narrative synthesis was conducted, as meta-analysis was precluded by the heterogeneity of digital interventions and outcome measures [19]. Two reviewers (AP, HH) read all papers, recorded key points and extracted data into a standardised Excel format. This recorded the specific outcome/theme addressed, for example, attitudes or digital health literacy. Studies were tabulated and analysed using content analysis, with relationships explored through grouping and textual descriptions. Inductive thematic analysis was carried out in the exploration of barriers and facilitators. The robustness of synthesis was evaluated through critical reflection on the synthesis process.

### Assessment of certainty and bias

Quality assessment followed Gough’s Weight of Evidence (WoE) framework (Appendix S4). Trustworthiness was judged using predefined quality criteria, and each study was assessed for appropriateness of design and relevance to the review questions [20–23]. Two reviewers (AP, HH) independently rated studies, resolving discrepancies through discussion. No study was excluded based on its rating, but higher-quality papers were given more weight in the synthesis.

**Table 1:** Summary of included studies.

Study characteristics				Population characteristics		Intervention	Where in the perioperative pathway?	Which theme was addressed?				Digital or health literacy measure used	Key insights	Overall quality assessment
Year	Author	Study design	Country; single or multi centre	Sample size (*n*); Age mean [SD] or median (range)	Surgical specialty	Digital tool		Digital literacy	Digital access	Attitudes	Barriers			
2023	Cuadra	Retrospective observational	USA; single centre	3537; 78 (IQR 75–82)	Oncology	Web-based tool (electronic geriatric rapid fitness assessment)	Preoperative	Yes	No	No	Yes	Indirectly assessed	Older age and frailty were associated with longer time to complete web-based assessment and need for assistance (*P* < .001).	Strong
2023	Shaikh	Retrospective observational	USA; multicentre	19 721; pre-waiver 71 (IQR 67–76), post-waiver 70 (IQR 66–74)	Hepatobiliary	Internet use, telemedicine	Outpatients	No	Yes	No	Yes	Not measured	Higher SVI associated with lower proportion of internet access (low SVI: 81.9% access, IQR 57.7–86.2, high SVI: 71.4% access, IQR 65.0–77.6; *P* < .001). Telemedicine use decreased with age (OR 0.68 aged 65–69 compared with age 18–64).	Strong
2023	Wang	Qualitative	China; single centre	25; 68 (33–83)	Orthopaedics	Smartphone application, telerehabilitation, educational resources and videos	Postoperative	No	No	Yes	Yes	Not measured	Information via app was more comprehensive and reliable; provided better access to support and communication with healthcare professionals; provided opportunity to share information/advice/experiences.	Strong
2022	Backman	Qualitative	Canada; multicentre	10 older adults, 8 caregivers; 65–85+	Orthopaedics	Web-based tool (*MyPath to Home*)	Postoperative	No	No	No	Yes	Not measured	Main barriers included a need for familiarity with computers, preference for direct verbal communication. Main enablers included ease of access to information and lack of concern using web-based service. Carers reported more facilitators than barriers compared to patients.	Strong
2020	Jonker	Prospective observational	Netherlands; single centre	151; 72.8 [5.4]	Oncology	Telemonitoring	Preoperative and postoperative	No	Yes	No	Yes	Not measured	Compared with participants, non-participants were significantly older (*P* = .01) and more often female (*P* = .0). Non-participants had a significantly higher ASA classification (*P* = .0), more polypharmacy (*P* = .0) and less social support (*P* = .01).	Strong
2017	Choi	Qualitative	USA; single centre	15; 67.6 [7]	Orthopaedics	Web-based tool (pictograph discharge instructions)	Postoperative	Yes	No	No	Yes	S-TOFHLA	Low health literacy participants valued a large font size, simple drawings and language. Included participants had mean S-TOFHLA 17.1.	Moderate
2023	Claesens	Mixed methods	Netherlands, Germany, Austria; multicentre	22; 70 [7]	Ophthalmology	Smartphone application, web-based tool (*Cataract Online Refraction Evaluation*)	Preoperative	No	Yes	Yes	Yes	Not measured	Most participants responded positively to the web-based test. Barriers included a lack of confidence, reliability of own device and preference for in-person hospital assessment which was seen as more trustworthy; 27% had carer assistance.	Moderate
2023	Poli	Post-trial analysis	Sweden; multicentre	498; 70 [0.32]	Orthopaedics, general surgery, breast, urology	Smartphone application	Postoperative	Yes	Yes	Yes	Yes	Self-reported digital skills	Those aged 75 years or older had a higher probability of being non-screened (odds ratio (OR) = 3.80, *P* < .05), non-recruited (OR = 12.09, *P* < .001) or a decliner (OR = 2.61, *P* < .05) versus those aged 65–69. Main reason for non-recruitment was technology-related, increasing with age.	Moderate
2023	Van der Storm	Retrospective observational	Netherlands; members of Dutch Stoma Association	1868; 67.5 [11.6]	Colorectal, urology	Internet use, smartphone applications	Postoperative	No	Yes	Yes	Yes	Not measured	39% used the internet at least once a month to search for stoma information; 32% never used the internet. Mobile tech experience: 3.2% none, 10% little, 20% excellent. Use of mobile apps 60%, but no medical apps. 14.6% had no mobile phone. 70.9% of adults over-80 had experience with mobile technology.	Moderate
2022	DeFrance	Retrospective observational	USA; multicentre	293; 66 (26–89)	Orthopaedic	Wearable technology, telemonitoring	Postoperative	No	Yes	Yes	Yes	Not measured	83.6% of patients were willing to wear a remote monitoring device. Individuals aged 60–69 years were 4x more likely to wear a monitoring device than individuals aged 80–89 years (*P* < .01). Those with prior wearable experience were 7x more likely to state they would wear again (*P* < .001).	Moderate
2022	Mirone	Prospective observational	Italy; single centre	697; 67.5 [5.3]	Urology	Smartphone application, email, phone calls	Postoperative	No	Yes	Yes	Yes	Not measured	Age 55–74 were highest users of telemedicine compared with all ages. Those >75 years more likely to never (34%) or rarely (37%) use telemedicine.	Moderate
2022	Saunders	Qualitative	Australia, single centre	9; 69 (53–70)	Orthopaedic	Web-based tool (*My Hip Journey*)*,* telerehabilitation, educational resources and videos	Preoperative and postoperative	No	No	Yes	Yes	Not measured	Web-based tools promoted engagement with surgical pathways, videos on postoperative topics such as wound care were reassuring and direct communication with the hospital team was appreciated. App was straightforward even for those without strong computer literacy.	Moderate
2021	De Looper	Prospective observational	Netherlands; multicentre	90; 69.9 [9.9]	Colorectal oncology	Internet use	Preoperative	Yes	Yes	No	No	3-item health literacy questionnaire	Patients’ health literacy was relatively high (mean 4.25, SD 0.71). Age was negatively related to OHIS (r = −0.29; *P* = <.005). Education level and OHIS were positively correlated (r = 0.37; *P* < .001). No significant correlation between OHIS and gender (r = 0.01; *P* = .91), frailty (r = −0.10; *P* = .35) and health literacy (r = 0.15; *P* = .14).	Moderate
2021	Huynh	Qualitative	USA; single centre	11; 67.7 [12.6]	Gastrointestinal oncology	Smartphone application *(MobiMD)*	Postoperative	No	Yes	Yes	Yes	Not measured	91% smartphone ownership, 45% had history of mobile health app use. Mostly positive perceptions of mobile health apps. Negative perceptions relating to lack of digital skills and lack of need.	Moderate
2019	Wieldraaijer	Prospective observational	Netherlands; multicentre	259; 67 [10.1]	Colorectal	Internet use	Postoperative	No	Yes	No	Yes	Not measured	69% of patients used the internet for information in 6-months after treatment. Patients younger than 65 years more frequently searched for information themselves [OR 1.66 (1.13–2.43)] and more often used the internet [OR 2.58 (1.66–3.99)] versus those older than 65 years. University educated more frequently searched for information themselves [OR 2.18 (1.24–3.82)] and used the internet [OR 3.26 (1.58–6.72)].	Moderate
2018	Dale	Retrospective observational	Norway; single centre	197; 67.3 [8.3]	Cardiothoracic	Internet use, e-learning, telemedicine	Postoperative	No	Yes	Yes	Yes	Not measured	58.4% cardiac surgery patients used the internet to find information about their cardiac disease, 30.5% found this information to be useful. Lower age was weakly associated with the variable ‘Digital communication could cover need for information and counselling’ (rs = −0.308; *P* < .001), but age did not come up as a predictor for attitudes toward using digital communication.	Moderate
2017	Burton	Retrospective observational	UK; multicentre	101; 82 (75–99)	Breast	Internet use	Postoperative	No	Yes	Yes	No	Not measured	Patients strongly preferred face to face information (81% preferred) and written formats (33%) over computer-based resources (2%). Only 29% of patients had their own computer with internet access.	Moderate
2024	Schulz	Randomised trial	Germany; single centre	160; 70.5 [14.2]	Dermatology	Web-based tool, educational resources and videos	Postoperative	No	No	Yes	No	Not measured	Digital-assisted informed consent outperformed written consent in most areas (*P* < .05). Highest satisfaction (4.8) and understanding (5.0) scores (1–7 Likert scale) in the ‘digitally assisted’ consent group with access to educational videos.	Weak
2022	Hise	Prospective observational	USA; single centre	104; 69.5 [7.2]	Vascular	Video conferencing	Outpatients	No	Yes	Yes	No	Not measured	104/185 veterans (56%) had a smartphone and were interested in care via video visit. Smartphone owners were younger than non-owners (67.3 vs. 73.2 years *P* < .001). Patients that had ≤2 comorbid diseases were more likely to own a smartphone (*P* = .049), those that had ≥5 comorbidities were less likely to own a smartphone (*P* = .041).	Weak
2022	Martin	Retrospective observational	UK; multicentre	1195; 67.4 (24–98)	Orthopaedic	Web-based tool, educational resources and videos, email	Preoperative and postoperative	No	Yes	No	No	Not measured	The majority of registered patients accessed their accounts. Adults aged 71–80 years spent longer on the program per access compared with those <50 years (*P* < .001); older age was associated with using a computer rather than smartphone or tablet (*P* < .001).	Weak
2020	Anderson	Retrospective observational	USA; multicentre	99; 72.7 [5.9]	Vascular	Internet use	Preoperative	No	Yes	No	No	Not measured	The internet was used as the primary source of information on open surgical repair and EVAR by 10% and 11% patients, respectively.	Weak
2019	Timmers	Randomised trial; multicentre	Netherlands; multicentre	114 intervention (daily access to app) v 99 control (basic access to app); 65.7 [7.7]	Orthopaedic	Smartphone application, educational resources and videos	Postoperative	No	Yes	Yes	No	Not measured	93/114 patients in the intervention group downloaded the postoperative care app. 75% participants used smartphones, 25% used tablets. Text-only items were accessed more frequently than videos. App users were more satisfied with information than control (*P* < .01).	Weak
2015	Yanes	Prospective observational	USA; single centre	116; 66.5 [14.8]	Urology, ophthalmology	Internet use	Preoperative	Yes	Yes	No	No	Self-reported confidence in English language	Patients with low confidence in their English abilities were less likely to have access to, and use the Internet before undergoing surgery (OR 2.9, *P* < .001). Older patients also use the Internet less often (OR 1.01, *P* < .001).	Weak
2010	Lim	Retrospective observational	UK; single centre	68; 67 (IQR 58–76)	Cardiac	Internet use	Preoperative	No	Yes	Yes	Yes	No	Most (61.8%) patients had access to the internet either at home or work. Twenty-nine (42.6%) patients used the web on a regular basis but 33 (48.5%) reported that they never used the internet.	Weak

Abbreviations: STOFHLA, Short Test of Functional Health Literacy in Adults; SVI, Social Vulnerability Index; OHIS, Online Health Information Seeking.

## Results

### Study characteristics

Twenty-four studies met the inclusion criteria. Study characteristics are shown in Table 1. All studies were published after 2010. In total, they include 29 067 participants. Mean age ranged from 65 to 82 years. Study designs included randomised trials (*n* = 2), prospective observational (*n* = 6), retrospective observational (*n* = 9), qualitative (*n* = 5), mixed methodology (*n* = 1) and *post-hoc* analysis (*n* = 1). Eleven were multi-centre studies. Research originated from: USA (*n* = 8); Netherlands (*n* = 6); UK (*n* = 3); Germany (*n* = 2); Australia, Austria, Canada, China, Italy, Norway and Sweden (*n* = 1). Surgical specialties included: orthopaedics (*n* = 8); urology (*n* = 4); oncology (*n* = 4); colorectal (*n* = 2); breast, ophthalmology, vascular (*n* = 2); cardiac, cardiothoracic, dermatology, general surgery and hepatopancreatobiliary (*n* = 1). Most studies focussed on the postoperative period (*n* = 13), followed by the preoperative period (*n* = 6), with smaller numbers exploring both (*n* = 3) or outpatient appointments only (*n* = 2).

Two studies had no exclusion criteria [24, 25]. Six papers specifically sought to investigate older adults [26–31], and one study specifically recruited patients living with frailty [29]. Two studies collected measures of frailty [27, 32] and one on level of cognition [31]. Three studies allowed participation of those with caregivers or family members to assist using the digital tool [26, 27, 29]. Study eligibility criteria that could limit the representation of older adults in the study population is detailed in Table 2.

**Table 2:** Eligibility criteria that potentially limits representation of older adults.

Exclusion domain	Eligibility criteria	*N* =	Studies
Language and literacy	Unable to speak or read primary study language	7	Claessens 2023, DeFrance 2022, de Looper 2021, Jonker 2020, Poli 2023, Timmers 2019, Wang 2023
	Not able to complete questionnaire themselves	2	Claessens 2023, Yanes 2015
	Not able to read	1	de Looper 2021
Digital access	No smart device (smart phone or tablet)	6	Claessens 2023, Hise 2022, Huynh 2021, Poli 2023, Timmers 2019, Wang 2023
	No internet access	3	Claessens 2023, Jonker 2020, Wang 2023
	No email	4	Dale 2018, Saunder 2022, Timmers 2019, van der Storm 2023
Cognition and capacity	Cognitive impairment or dementia	5	Backman 2022, Choi 2016, de Looper 2021, Poli 2023, Schulz 2024
	Lacking capacity to personally consent	3	de Looper 2021, Schulz 2024, Wang 2023
	Major psychiatric illness	1	Schulz 2024
Function	Non ambulatory or not able to complete rehabilitation	2	Jonker 2020, Wang 2023
Health	No frailty score available	1	Cuadra 2023
	Visual or hearing impairment	2	Choi 2016, Jonker 2020
	Life expectancy <12 months	1	Backman 2022

### Digital and health literacy

No studies used a validated digital literacy measure. In a prospective cohort postoperative telemonitoring in older adults after cancer surgery, 12 of 86 non-participants cited perceived digital illiteracy or inability to use electronic devices or mobile applications as their primary reasons for declining to participate [27]. In the ‘Mobile Phone in Recovery after Ambulatory Surgery’ (MIRAS) trial, low self-reported confidence in various digital skills was associated with declining to participate [31]. Six studies used surrogate markers of digital skills, including independently completing online tasks [29, 33], connecting to an application [34, 35] and previous mobile health application experience [36, 37]. In a web-based preoperative geriatric assessment, 48% of older adults required assistance, with younger age and lower frailty scores associated with independent use [29]. When using a web-based eye test, almost 27% of participants were assisted by a relative [33]. A survey of Danish stoma patients demonstrated that more than 70% of adults over 80 years had at least moderate mobile phone experience, although participants were required to have and use email to take part [36].

Two studies assessed health literacy [28, 32]. The ‘Short Test of Functional Health Literacy in Adults’ (S-TOFHLA), a validated 40-item questionnaire, identified older adults with lower literacy levels for inclusion in a qualitative study on web-based pictographs for hip replacement surgery discharge information [28]. A brief three-item health literacy tool was used in a prospective study of online health information-seeking before preoperative colorectal cancer clinics; increasing age, but not health literacy or frailty, was associated with reduced online information-seeking [32].

Education level was reported in 12 studies, though definitions varied [25, 28, 30–32, 34–36, 38–41]. Most categorised education as primary, secondary or tertiary; two reported on participants with no formal education [35, 41]. Two studies examined relationships between education and digital technology use, both finding higher education associated with greater internet use and online information-seeking [32, 40]. University-educated participants were three times more likely to use the internet (OR 3.08, CI 1.69–5.62) and twice as likely to do so independently (OR 2.18, CI 1.24–3.82) [40].

### Access to digital technology and tools

Seventeen studies looked at the use of digital tools. These were varied, including web-based applications [26, 28, 29, 33, 42–44], mobile applications [25, 31, 35–37, 41], telemedicine or telerehabilitation [27, 43, 45], wearable technology [39, 46] and video conferencing [34].

Several studies collected data on access to digital devices [25, 31, 33–37, 42, 46]. This included the availability, types and use of devices. Some studies reported the number of participants who owned devices but had excluded people who did not have digital devices or access, limiting the generalisability of these findings [33, 34, 36, 37]. Exclusion from participation through digital exclusion was common as shown in Table 2. In the *post-hoc* analysis of the MIRAS trial, over 70% of those not recruited were excluded due to lacking access to an internet-ready mobile phone, introducing bias. Almost 90% of the excluded group were over 70 years old [31]. In studies that compared older and younger adults, older adults were less likely to have used wearable technology, remote monitoring or telemedicine [25, 45, 46], used the internet less [40] and favoured computer use instead of mobile phones for web access [42].

Use of the internet by older adults varied. Eleven studies examined internet use as a primary [24, 27, 32] or secondary objective [30, 34, 36, 38–40, 45, 47]. Access to the internet was reported in two studies [27, 30]. One found that 10% of older adults screened for inclusion were not eligible due lack of internet access [27]. The second, a survey on information preferences for women over 75 years, found that nearly one-third owned a computer with internet access. However, one quarter of participants did not have internet access, and nearly two-thirds were ‘very’ or ‘somewhat unlikely’ to use the internet for information about their cancer [30]. Two studies reported 10% or less of older adults used the internet for information [30, 47]. Others found a mixed picture with 27% using the internet frequently but almost half (48%) not using it at all [32]. Some studies reported between half [39] and two-thirds of participants using the internet for information [40], but in the former study, an email address was required to participate. Importantly, internet access did not always translate to internet use. For example, a survey of cardiac surgery patients’ online behaviours found that while over 60% had access to the internet at home or work, and around 40% used the internet regularly, almost half (48%) never used the web [24].

Notably, for studies creating or implementing a new digital tool only, one reported codesign prior to implementation with older adults [26]. Five others collected patient feedback on clinician-designed [28, 37, 41, 43] or third-party designed digital tools [33].

### Attitudes toward digital perioperative interventions

Fourteen studies reported on attitudes of older adults towards perioperative digital tools. This was measured through patient interviews [33, 37, 43], surveys and questionnaires [25, 30, 33–36, 39, 44–46], concordance with interventions [35, 42] and reasons for non-participation [31].

Most studies reported positive attitudes towards digital interventions. Participants were interested in using mobile applications [33, 37, 41], telemedicine [25, 34] and wearable technology [46]. Many studies reported that digital tools and information sources were considered trustworthy and reliable [24, 33, 34, 41]. Two studies reported that digital tools improved access to health care by facilitating remote contact with healthcare professionals [41] or additional support and guidance [39]. Across a number of studies, the potential for patient empowerment through digital interventions was highlighted. Perioperative applications were reported to facilitate self-management, improve motivation and engagement with rehabilitation and promote lifestyle changes [33, 37, 39, 43]. Patients particularly liked the forum used in one application that allowed sharing of patient experiences at different stages of recovery and fostered a feeling of community [41].

Fewer studies reported downsides. In one mixed-methods study, participants expressed concern that increasing digital usage could detract from important human interactions during postoperative recovery. Contact with their healthcare team was an opportunity to express gratitude and also to provide feedback on their experiences. While some participants considered the applications trustworthy, others thought conventional hospital appointments and assessments were the gold standard [33]. Some older adults felt that a digital tool during their surgical journey was unnecessary, as they had been able to track their health adequately without the use of technology [37].

There were mixed results regarding older adults’ information and communication preferences. This ranged from a preference for face-to-face or written communication [26, 30] to a preference for internet or digital platforms over traditional formats [36, 41, 43]. The strongest opinion was expressed in one study of women aged over 75 years undergoing breast surgery of whom over 80% wanted to receive information about their diagnosis and treatment face-to-face from their doctor with only 2% preferring information from the internet [30]. Two studies demonstrating a preference for digital platforms, gathered patient views after trying a novel telerehabilitation application [41, 43]. The third study surveyed patients regarding the use of web-based information or a mobile application for stoma-care but did not offer face-to-face communication as an option. Patients expressed a preference for web-based information, but mobile applications were less popular than paper options [36].

### Barriers and facilitators

Sixteen studies described barriers and facilitators to the use of digital interventions in the older perioperative population [24–29, 31, 33, 36, 37, 39–41, 43, 45, 46]. Four themes were identified.

#### Digital exclusion

Digital exclusion, namely a lack of access, affordability and ability to use digital technologies and devices was identified as a barrier in several studies [25–27, 30, 31, 37]. To overcome this, one study provided the option to complete the online form using a computer at the clinic [29]. No studies provided devices for participants to use at home. A USA medicare database study reported that the most vulnerable communities, defined by the Social Vulnerability Index, had 10% less internet access than the least vulnerable [45]. In contrast, greater familiarity with digital technology was associated with higher levels of engagement [36, 41, 46].

Digital illiteracy, defined as a lack of digital skills or lack of confidence using digital tools was also cited as a barrier. Participants reported a perceived inability to use electronic devices and mobile applications and a dislike of technology [27, 31, 37].

#### Device usability

Several studies suggested that ease of use of digital interventions is a facilitator to digital usage [26, 28, 33, 37, 43]. Applications with features adapted for sensory impairments, for example, larger images and adjustable text size, were seen as beneficial [28]. Individuals with lower levels of literacy found visual information, using pictures or video, helpful [28, 41]. Feedback also suggested integrated audio recording of text-based medical terminology helped with pronunciation [28]. Older adults found videos useful as part of online educational platforms. They reported improved confidence through watching other patients complete physiotherapy exercises, felt that being able to rewatch videos helped reinforce routines, and suggested that instructional videos on how to use apps might be useful [41]. Older adults wanted perioperative digital tools to include medication management tools and the facility to send photos to enable digital postoperative wound review, thus reducing attendance at clinics [37].

#### Training and caregiver support

In three studies, family, friends or a primary caregiver facilitated the use of digital interventions [27, 33, 37]. All were conducted in a population that owned their own smartphones. Older adults suggested that involving their families would be helpful when using smartphone applications [41]. Participants with a lack of experience with digital technology may require more training [37, 43], which could add burden to clinical workflow. However, this investment in time may improve downflow workstreams. Time required for training was not quantified as an outcome by any study.

#### Patient factors

Older age [27, 29, 31, 32, 34, 35, 39, 45, 46], comorbidity [17, 31, 36], lower educational level [32, 41] and greater social vulnerability [27, 45] were associated with reduced use or acceptability of digital tools, increased support needs, non-participation or attrition from studies. Those living with frailty took longer to complete a web-based assessment and were more likely to require support [29]. One study specifically implementing strategies to improve inclusivity for older adults, including face-to-face contact, clearly written study information, a case-manager available by telephone and involvement of family members in recruitment, found that non-participants were more likely to be older [27]. Self-selection for study participation may drive exclusion and contribute to inequalities among older people in digital health research [41].

For some older adults, digital technology helped to reduce travel time and avoid issues with traffic and parking, which was seen as a benefit [33, 37, 41]. When individuals trusted that their data was secure and information was reliable, they were more likely to use remote monitoring [46] or applications [37]. When healthcare professionals were more easily accessible through a digital tool, this was seen as a facilitator. Feedback from patients using a WeChat-based platform in their postoperative recovery, reported that they were able to create rapport with healthcare professionals and supportive relationships with other patients through the forums and communication channels. However, it was also suggested that the 24/7 availability of a clinician would be unsustainable after the study and defined online ‘office hours’ might offer a pragmatic solution [41].

One study specifically looked at barriers and facilitators and found several conflicting themes possibly reflecting the heterogeneity within this population. Patients reported more barriers than enablers to using digital technology, while carers identified more enablers than barriers [26].

### Quality assessment

WoE framework was used to assess quality (averaged ratings in Table 1; full framework in Appendix S4). It comprises four components:

1. WoE A: Trustworthiness—overall rigour and technical quality.
2. WoE B: Appropriateness—suitability of study design for the review questions.
3. WoE C: Relevance—relevance of study focus to the review questions.
4. Totality of evidence—consideration of the strength of the evidence base overall.

Methodological quality (WoE A) varied, with most studies rated moderate. Both randomised trials were considered weak due to small sample sizes, concerns around blinding and control groups and limited relevance to review questions. Selection bias was frequent, with convenience sampling and exclusion of people with sensory, physical or cognitive impairment, and the digitally excluded. Several study populations were predominantly male and of white ethnicity, limiting generalisability to the wider perioperative population, which is more diverse and often comprises patients living with multimorbidity and frailty. Methodological heterogeneity was substantial. Appropriateness and relevance to directly answer the review questions was also variable, with most studies receiving moderate or weak ratings, as the review questions were often indirectly assessed rather than serving as primary outcomes.

## Discussion

Given rising interest in perioperative digital tools and an ageing surgical population, this review highlights how digital and eHealth literacy and attitudes toward digital devices can inform co-development of such tools. Older adults may benefit from tools that reduce clinic burden, support self-management, build confidence during prehabilitation and rehabilitation, provide prompts and reminders and enable early communication to prevent readmission. Many perioperative digital innovations—from smartphone and web-based applications to telerehabilitation, wearables and remote monitoring for virtual wards—are emerging [48], yet few have been trialled in older adults. Little is known about how these innovations can optimise care for those living with frailty or requiring caregiver support to access or use digital technology.

Research in this area is limited by bias from exclusion of patients without digital access. Many studies excluded those unable to use mobile phones, smart technology, internet or email. This is despite 18% of over 65s and 29% of over 75s in the UK not using the internet, 77% of people with no basic digital skills being over 65 and 69% of those without such skills having a disability or impairment [49, 50]. These excluded groups are more likely to live with multimorbidity and frailty and may benefit most from accessible digital technology due to high appointment burden, cost and travel time. Reporting of health literacy, digital literacy and digital inclusion was variable and, in most cases, lacked validated measures such as the eHEALS, Mobile Device Proficiency Questionnaire (MDPQ) and DHLI [51]. Higher education level (reported by half of studies) is associated with better digital health literacy but only represents one aspect of the many complex determinants [15].

Several studies reported digital access and use as secondary outcomes, mainly through surveys focused on technology relevant to the tool being tested, rather than broader digital inclusion. Studies trialling novel tools tended to show stronger preferences for digital over non-digital modalities compared with studies examining general patient preferences, information-seeking or communication. Studies of existing tools [25, 28, 29, 31, 33–35, 37, 41–44] generally showed positive attitudes and feasibility, whereas studies informing the design of digital interventions more often reported negative attitudes [24, 26, 27, 30, 32, 36, 39, 40, 45–47]. Only one digital tool was codesigned with patients.

Barriers and facilitators identified here align with previous work in older surgical and non-surgical populations [17, 52, 53]. Poorly tailored tools, limited access, high device costs, unreliable internet and reliance on family support remain key barriers [53]. Older adults preferred tools that enhanced, rather than replaced, access to clinicians. In-person consultations were valued for diagnosis and treatment planning. Digital options that reduced unnecessary appointments—such as sending photos for wound review—were viewed positively, but personal interaction and relationship-building remained important.

Narrative synthesis was required due to study and outcome heterogeneity, but remains a limitation of this review. Following best practice, data collection was predefined to improve objectivity, and two researchers contributed to all stages to reduce subjective interpretation. Many pilot and feasibility studies were excluded; these small, purposively sampled studies (often <50 participants) focused on concordance and adherence rather than measuring digital or health literacy. Attitudes were frequently inferred from proxy markers such as logins or time online rather than direct assessment. We recognise that family members and caregivers play a crucial role in the care of older people through the perioperative pathway and should be included in codesign of future digital tools. Despite not specifically including search terms to capture carers, this theme emerged in the barriers and facilitators. While we chose to focus this review on older surgical patients, a group traditionally excluded from digital health research, the role and views of their carers could form the basis of future research.

## Conclusion

Across surgical specialties and perioperative pathways, there is a clear and growing interest in the development and use of digital tools for older adults undergoing surgery. However, the current evidence base remains limited in its generalisability, through use of heterogeneous methodologies; inconsistent measurement of digital literacy; and selection bias arising from implicit and explicit exclusion of those with sensory, cognitive or functional impairments, frailty, multimorbidity and the digitally excluded. Although many studies report positive findings, few have been implemented at scale or with sufficient attention to digital inclusion. Robust research is needed to characterise eHealth literacy, digital inclusion and attitudes towards digital innovations in this diverse population. Embedding meaningful codesign with older adults as key stakeholders is essential to ensure that future perioperative digital interventions are developed using appropriate modalities, are accessible and equitable and can be widely implemented.

## Supplementary Material

Supplementary_materials_afag165
