# Supplementary material for: What is known about digital literacy, digital inclusion and attitudes to digital health tools among older adults undergoing surgery? A systematic review and narrative synthesis
Source: Age Ageing. 2026 Jun 8;55(6):afag165. doi: 10.1093/ageing/afag165 (PMC13245732; doi:10.1093/ageing/afag165)
Supplement: Supplementary_materials_afag165 [file supplementary_materials_afag165.zip › Supplementary_materials_afag165_Appendix 4.docx]

**Gough Weight of Evidence (WoE) Framework for quality assessment**

**Table 4A: The judgement of overall weight of evidence is based on the assessment made for each criteria A-C (trustworthiness, appropriateness, relevance).**

|  | **Strong** | **Moderate** | **Weak** |
| --- | --- | --- | --- |
| **Weight of Evidence A**  **TRUSTWORTHINESS**  *The trustworthiness of the results judged by the quality of the study within the norms of undertaking the particular type of research design used in the study (methodological quality).* | - Clear focused research question with research design appropriate to aims  - Accurate and understandable reporting of data with appropriate statistical analysis  - No significant sources of bias or confounding; if minor factors present these are identified and addressed  - Conclusions flow from findings and are proportionate to method | - Research question identified with research design mostly appropriate to address aims  - Satisfactory reporting of data and statistical analysis  - Minor to moderate bias or confounding factors which may not be fully identified and addressed  - Conclusions mostly flow from findings and are proportionate to method | - Unclear research question and/or research design mostly inappropriate to address aims  - Poor reporting of data with less appropriate statistical analysis  - Moderate to significant bias or confounding factors which are not identified or addressed  - Conclusions flow poorly from findings and are not proportionate to method |
| **Weight of Evidence B**  **APPROPRIATENESS**  *The appropriateness of the use of that study design for addressing the systematic review research question (methodological relevance).* | **What is known about e-health/digital literacy, health literacy or availability of internet/electronic devices/digital tools for older adults undergoing elective surgery?** | | |
|  | Large observational studies including cross sectional, databases and cohort studies that directly assess the above. | Other study designs directly assessing the above. | Study designs not directly assessing the above or using surrogate markers (e.g. internet use, compliance with using device). |
|  | **What are the attitudes of older adults undergoing elective surgery towards digital interventions? What barriers and facilitators exist towards use of perioperative digital tools by older adults?** | | |
|  | Inductive research designs interpreting views reported by older adults undergoing surgery or directly exploring barriers to participation | Deductive research designs interpreting views reported by older adults undergoing surgery or indirectly exploring barriers to participation | Research which reports acceptability of or engagement with digital tools as proxy for attitudes; barriers and facilitators not assessed or explored. |
| **Weight of Evidence C**  **RELEVANCE**  *The appropriateness of focus of the research for answering the review question.* | Meets ≥2 of:  - Research specifically on older adults undergoing surgery  - Objectives and outcomes directly relevant to the review questions  - Representative of the typical perioperative population (i.e. little selection bias, diverse patient group, mix of surgical specialties) | Meets 1 of strong criteria or partly meets 2. | Meets none of the strong criteria or partly meets 1. |

**Table 4B: Weight of evidence breakdown by study**

| **Study** | **WoE A**  **Trustworthiness** | **WoE B**  **Appropriateness** | **WoE C**  **Relevance** | **Overall Judgement** |
| --- | --- | --- | --- | --- |
| Anderson, 2020 | Weak | Weak | Weak | Weak |
| Backman, 2022 | Strong | Strong | Strong | Strong |
| Burton, 2017 | Moderate | Moderate | Strong | Moderate |
| Choi, 2016 | Moderate | Moderate | Strong | Moderate |
| Claessens, 2023 | Moderate | Moderate | Moderate | Moderate |
| Cuadra, 2024 | Strong | Weak | Strong | Strong |
| Dale, 2018 | Weak | Moderate | Moderate | Moderate |
| DeFrance, 2022 | Moderate | Weak | Moderate | Moderate |
| de Looper, 2021 | Moderate | Moderate | Weak | Moderate |
| Hise, 2022 | Weak | Weak | Moderate | Weak |
| Huynh, 2021 | Moderate | Moderate | Moderate | Moderate |
| Jonker, 2020 | Strong | Moderate | Strong | Strong |
| Lim, 2010 | Weak | Weak | Weak | Weak |
| Martin, 2022 | Weak | Weak | Weak | Weak |
| Mirone, 2022 | Moderate | Moderate | Moderate | Moderate |
| Poli, 2021 | Moderate | Moderate | Strong | Moderate |
| Saunders, 2021 | Moderate | Strong | Moderate | Moderate |
| Schulz, 2024 | Weak | Weak | Weak | Weak |
| Shaikh, 2023 | Strong | Strong | Moderate | Strong |
| Timmers, 2019 | Weak | Weak | Weak | Weak |
| van der Storm, 2023 | Moderate | Moderate | Moderate | Moderate |
| Wang, 2023 | Weak | Strong | Strong | Strong |
| Wieldraaijer, 2019 | Moderate | Weak | Moderate | Moderate |
| Yanes, 2015 | Weak | Moderate | Weak | Weak |
