# Supplementary material for: What is known about digital literacy, digital inclusion and attitudes to digital health tools among older adults undergoing surgery? A systematic review and narrative synthesis
Source: Age Ageing. 2026 Jun 8;55(6):afag165. doi: 10.1093/ageing/afag165 (PMC13245732; doi:10.1093/ageing/afag165)
Supplement: Supplementary_materials_afag165 [file supplementary_materials_afag165.zip › Supplementary_materials_afag165_Appendix 3.docx]

| PICO table of inclusion and exclusion criteria for study selection | | |
| --- | --- | --- |
| **Domain** | **Inclusion Criteria** | **Exclusion Criteria** |
| **Population** | Clinical study conducted on human populations with mean age over 65 | *In vivo* studies  *Ex vivo* studies  Animal studies |
| **Intervention** | Any surgical procedure  Any digital tool or intervention used by patients during the perioperative period | Patients undergoing non-surgical procedures, or studies describing surgical technique  Telephone-only intervention |
| **Comparison** | NA | N/A |
| **Outcome** | Health literacy  Digital or eHealth literacy  Availability of digital tools  Attitudes towards digital interventions  Barriers and facilitators to usage of digital interventions | Studies not describing digital or health literacy, attitudes toward or availability of digital tools, or barriers and facilitators to usage of digital interventions |
| **Study Type** | Primary studies written in English with full-text available  Experimental, quasi-experimental and observational studies, including quality improvement programmes and case series | Case reports, abstracts, reviews, editorials, letters, feasibility studies, pilot studies  Studies not written in English |
