# Supplementary material for: What is known about digital literacy, digital inclusion and attitudes to digital health tools among older adults undergoing surgery? A systematic review and narrative synthesis
Source: Age Ageing. 2026 Jun 8;55(6):afag165. doi: 10.1093/ageing/afag165 (PMC13245732; doi:10.1093/ageing/afag165)
Supplement: Supplementary_materials_afag165 [file supplementary_materials_afag165.zip › Supplementary_materials_afag165_Appendix 2.docx]

**Search Strategy**

|  | **Medline (PubMed)** | **Ovid (Embase)** | **CINHAL** | **Cochrane** |
| --- | --- | --- | --- | --- |
| 1 | exp Aged/ | aged/ | (MH "Aged+") | Exp Aged/ |
| 2 | Geriatrics/ | geriatrics/ | (MH "Geriatrics+") | Exp Geriatrics/ |
| 3 | Aging/ | aging/ or cognitive aging/ or healthy aging/ | (MH "Gerontologic Care") | Geriatric Assessment/ |
| 4 | Frailty/ | frailty/ | (MH "Frailty Syndrome") | Geriatric Anaesthesia/ |
| 5 | Geriatric Assessment/ | old*.tw,kf. | (MH "Aging") | Aging/ |
| 6 | old*.tw,kf. | geriatric*.tw,kf. | (MH "Geriatric Assessment+") | Frailty/ |
| 7 | geriatric*.tw,kf. | elder*.tw,kf. | TI old* OR AB old* | (old*):ti,ab,kw |
| 8 | elder*.tw,kf. | 1 or 2 or 3 or 4 or 5 or 6 or 7 | TI geriatric* OR AB geriatric* | (geriatric*):ti,ab,kw |
| 9 | 1 or 2 or 3 or 4 or 5 or 6 or 7 or 8 | perioperative period/ or intraoperative period/ or perioperative care/ or postoperative care/ or preoperative period/ | TI elder* OR AB elder* | (elder*):ti,ab,kw |
| 10 | Perioperative period/ or intraoperative period/ or postoperative period/ or preoperative period/ | perioperative medicine/ | S1 OR S2 OR S3 OR S4 OR S5 OR S6 OR S7 OR S8 OR S9 | #1 OR #2 OR #3 OR #4 OR #5 OR #6 OR #7 OR #8 OR #9 |
| 11 | Perioperative medicine/ | preop*.tw,kf. | (MH "Perioperative Medicine") | Exp Perioperative period/ |
| 12 | exp Perioperative care/ | pre-op*.tw,kf. | (MH "Perioperative Care+") OR (MH "Enhanced Recovery After Surgery") OR (MH "Intraoperative Care+") OR (MH "Postoperative Care+") OR (MH "Preoperative Care+") OR (MH "Postoperative Period") OR (MH "Preoperative Period+") OR (MH "Post Anesthesia Care") | Perioperative medicine/ |
| 13 | preop*.tw,kf. | periop*.tw,kf. | TI peri#op* OR AB peri#op | Exp Perioperative care/ |
| 14 | pre-op*.tw,kf. | peri-op*.tw,kf. | TI pre#op* OR AB pre#op* | Perioperative nursing/ |
| 15 | periop*.tw,kf. | postop*.tw,kf. | TI post#op* OR AB post#op* | (pre*op*):ti,ab,kw |
| 16 | peri-op*.tw,kf. | post-op*.tw,kf. | TI intra#op* OR AB intra#op* | (peri*op*):ti,ab,kw |
| 17 | postop*.tw,kf. | intraop*.tw,kf. | TI surger* OR AB surger* | (intra*op*):ti,ab,kw |
| 18 | post-op*.tw,kf. | intra-op*.tw,kf. | TI surgical* OR AB surgical* | (post*op*):ti,ab,kw |
| 19 | intraop*.tw,kf. | surger*.tw,kf. | S11 OR S12 OR S13 OR S14 OR S15 OR S16 OR S17 OR S18 | (surger*):ti,ab,kw |
| 20 | intra-op*.tw,kf. | surgical.tw,kf. | (MH "Health Literacy") | (surgical*):ti,ab,kw |
| 21 | surger*.tw,kf. | 9 or 10 or 11 or 12 or 13 or 14 or 15 or 16 or 17 or 18 or 19 or 20 | (MH "Computer Literacy") | #11 OR #12 OR #13 OR #14 OR #15 OR #19 OR #20 |
| 22 | surgical.tw,kf. | health literacy/ | (MH "Information Literacy+") | Health Literacy/ |
| 23 | 10 or 11 or 12 or 13 or 14 or 15 or 16 or 17 or 18 or 19 or 20 or 21 or 22 | information literacy/ | (MH "Digital Health+") | Information Literacy/ |
| 24 | Health Literacy/ | internet literacy/ | MH "Literacy") OR (MH "Illiteracy") | Computer Literacy/ |
| 25 | Information Literacy/ | computer literacy/ | TI "*health litera*" OR AB "*health litera*" | Digital Health/ |
| 26 | Computer Literacy/ | ehealth literacy/ | TI "computer litera*" OR AB "computer litera*" | Literacy/ |
| 27 | Digital Health/ | literacy/ | TI "digi* litera*" OR AB "digi* litera*" | (*health NEXT literacy):ti,ab,kw |
| 28 | Literacy/ | “*health literacy”.tw,kf. | TI "digi* skill*" OR AB "digi* skill*" | (computer NEXT litera*):ti,ab,kw |
| 29 | “*health literacy”.tw,kf. | “computer litera*”.tw,kf. | TI "digi* health*" OR AB "digi* health*" | (digi* NEXT litera*):ti,ab,kw |
| 30 | “computer litera*”.tw,kf. | “digi* litera*”.tw,kf. | TI e#health OR AB e#health | (digi* NEXT skill*):ti,ab,kw |
| 31 | “digi* litera*”.tw,kf. | “digi* skill*”.tw,kf. | TI m#health OR AB m#health | (digi* NEXT health*):ti,ab,kw |
| 32 | “digi* skill*”.tw,kf. | “digi* health*”.tw,kf. | TI "mobile health*" OR AB "mobile health*" | (e*health):ti,ab,kw |
| 33 | “digi* health*”.tw,kf. | ehealth.tw,kf. | S20 OR S21 OR S22 OR S23 OR S24 OR S25 OR S26 OR S27 OR S28 OR S29 OR S30 OR S31 OR S32 | (m*health):ti,ab,kw |
| 34 | ehealth.tw,kf. | e-health.tw,kf. | (MH "Digital Technology") | ("mobile health"):ti,ab,kw |
| 35 | e-health.tw,kf. | mhealth.tw,kf. | TI "digi* tech*" OR AB "digi* tech*" | #22 OR #23 OR #24 OR #25 OR #26 OR #27 OR #28 OR #29 OR #30 OR #31 OR #32 OR #33 OR #34 |
| 36 | mhealth.tw,kf. | m-health.tw,kf. | (MH "Internet+") | Digital Technology/ |
| 37 | m-health.tw,kf. | “mobile health”.tw,kf. | TI "internet" OR AB "internet" | (digi* NEXT tech*):ti,ab,kw |
| 38 | “mobile health”.tw,kf. | 22 or 23 or 24 or 25 or 26 or 27 or 28 or 29 or 30 or 31 or 32 or 33 or 34 or 35 or 36 or 37 | (MH "Computers and Computerization") | Exp Internet/ |
| 39 | 24 or 25 or 26 or 27 or 28 or 29 or 30 or 31 or 32 or 33 or 34 or 35 or 36 or 37 or 38 | digital technology/ | (MH "Cellular Phone+") OR (MH "Smartphone") | ("internet"):ti,ab,kw |
| 40 | Digital technology/ | digital health technology/ | TI computer OR AB computer | Computers/ |
| 41 | “digi* tech*”.tw,kf. | “digi* tech*”.tw,kf. | TI laptop OR AB laptop | Exp Cell Phone/ |
| 42 | exp Internet/ | internet/ | (TI digi* N5 tablet OR AB digi* N5 tablet) OR (TI computer* N5 tablet OR AB computer* N5 tablet) OR (TI smart N5 tablet OR AB smart N5 tablet) | (computer*):ti,ab,kw |
| 43 | internet.tw,kf. | internet.tw,kf. | TI "smart#phone*" OR AB "smart#phone*" | (laptop*):ti,ab,kw |
| 44 | Computers/ | computer/ or computer system/ or digital computer/ or personal computer/ or personal digital assistant/ | TI "cell#phone*" OR AB "cell#phone*" | (tablet* near/5 computer*):ti,ab,kw |
| 45 | exp cell phone/ | mobile phone/ or smartphone/ | TI "mobile phone*" OR AB "mobile phone*" | (tablet* near/5 digi*):ti,ab,kw |
| 46 | computer*.tw,kf. | tablet computer/ | TI "text messag*" OR AB "text messag*" | (tablet* near/5 "smart"):ti,ab,kw |
| 47 | laptop*.tw,kf. | wearable computer/ or wearable device/ or smart watch/ | TI email* OR AB email* | (smart*phone*):ti,ab,kw |
| 48 | ("tablet*" adj5 "computer*").tw,kf. | computer*.tw,kf. | (MH "Telehealth+") | (cell NEXT phone*):ti,ab,kw |
| 49 | ("tablet*" adj5 "digi*").tw,kf. | laptop*.tw,kf. | TI "tele#health*" OR AB "tele#health*" | (mobile NEXT phone*):ti,ab,kw |
| 50 | ("tablet*" adj5 "smart").tw,kf. | smartphone*.tw,kf. | TI "tele#medicine" OR AB "tele#medicine" | (text NEXT messag*):ti,ab,kw |
| 51 | smartphone*.tw,kf. | "mobile phone*".tw,kf. | (MH "Telenursing") | (email*):ti,ab,kw |
| 52 | “mobile phone*”.tw,kf. | ("tablet*" adj5 "computer*").tw,kf. | TI "telenursing" OR AB "telenursing" | Exp Telemedicine/ |
| 53 | “text messag*”.tw,kf. | ("tablet*" adj5 "digi*").tw,kf. | (MH "Electronic Bulletin Boards") OR (MH "Email") OR (MH "Instant Messaging") OR (MH "Text Messaging+") OR (MH "Videoconferencing+") OR (MH "Wireless Communications") | (telemedicine):ti,ab,kw |
| 54 | email*.tw,kf. | ("tablet*" adj5 "smart").tw,kf. | (MH "Wearable Sensors+") | (telehealth*):ti,ab,kw |
| 55 | Telemedicine/ | text messaging/ | TI "smart*watch*" OR AB "smart*watch*" | MeSH descriptor: [Remote Sensing Technology] this term only |
| 56 | telemedicine.tw,kf. | “text messag*”.tw,kf. | TI "fit* track*" OR AB "fit* track*" | MeSH descriptor: [Wireless Technology] this term only |
| 57 | telehealth.tw,kf. | e-mail/ | TI "wearable tech*" OR AB "wearable tech*" | MeSH descriptor: [Wearable Electronic Devices] explode all trees |
| 58 | Remote sensing technology/ | email*.tw,kf. | TI "smart home" OR AB "smart home" | (smart NEXT watch*):ti,ab,kw |
| 59 | Wireless technology/ | telemedicine/ | TI "smart tech*" OR AB "smart tech*" | (fit* NEXT track*):ti,ab,kw |
| 60 | exp Wearable Electronic Devices/ | telemedicine.tw,kf. | TI "video call*" OR AB "video call*" | (wearable NEXT tech*):ti,ab,kw |
| 61 | “smart watch*”.tw,kf | telehealth/ | TI "video app*" OR AB "video app*" | (smart NEXT home):ti,ab,kw |
| 62 | “fit* track*”.tw,kf. | telehealth.tw,kf. | TI "video conferenc*" OR AB "video conferenc*" | (smart NEXT tech*):ti,ab,kw |
| 63 | “wearable tech*”.tw,kf | “smart watch*”.tw,kf | TI "virtual" OR AB "virtual" | MeSH descriptor: [Mobile Applications] this term only |
| 64 | “smart home*”.tw,kf. | “fit* track*”.tw,kf. | TI "web-based" OR TI "web based" OR AB "web-based" OR AB "web based"  TI "web-based" OR TI "web based" OR AB "web-based" OR AB "web based" OR TI "app based" OR AB "app based" OR TI "app-based" OR AB "app-based" | (tech* NEXT app*):ti,ab,kw |
| 65 | “smart tech*”.tw,kf. | “wearable tech*”.tw,kf | (MH "Virtual Reality+") | (digi* NEXT app*):ti,ab,kw |
| 66 | Mobile applications/ | “smart home*”.tw,kf. | TI "virtual reality" OR AB "virtual reality" | (mobile NEXT app*):ti,ab,kw |
| 67 | “tech*” “app*”.tw,kf. | “smart tech*”.tw,kf. | TI "virtual medicine" OR AB "virtual medicine" | MeSH descriptor: [Videoconferencing] this term only |
| 68 | “digi*” “app*”.tw,kf. | mobile application/ or mobile health application/ | S34 OR S35 OR S36 OR S37 OR S38 OR S39 OR S40 OR S41 OR S42 OR S43 OR S44 OR S45 OR S46 OR S47 OR S48 OR S49 OR S50 OR S51 OR S52 OR S53 OR S54 OR S55 OR S56 OR S57 OR S58 OR S59 OR S60 OR S61 OR S62 OR S63 OR S64 OR S65 OR S66 OR S67 | (video NEXT call*):ti,ab,kw |
| 69 | “Mobile app*”.tw,kf. | “tech*” “app*”.tw,kf. | (MH “Digital Divide”) | (video NEXT app*):ti,ab,kw |
| 70 | Videoconferencing/ | “digi*” “app*”.tw,kf. | (MH "Internet Access") | (video NEXT conferenc*):ti,ab,kw |
| 71 | “Video call”.tw,kf. | “mobile app*”.tw,kf. | (MH "Health Services Accessibility+") | MeSH descriptor: [Virtual Reality] this term only |
| 72 | “Video conferencing”.tw,kf. | virtual reality/ | (MH "Health Care Delivery") | (“virtual”):ti,ab,kw |
| 73 | Virtual reality/ | virtual reality system/ | TI "digi* divi*" OR AB "digi* divi*" | (web* NEXT based):ti,ab,kw |
| 74 | virtual.tw,kf. | virtual.tw,kf. | TI access* OR AB access* | (app* NEXT based):ti,ab,kw |
| 75 | web-based.tw,kf. | web-based.tw,kf. | TI avail* OR AB avail* | #36 OR #37 OR #38 OR #39 OR #40 OR #41 OR #42 OR #43 OR #44 OR #45 OR #46 OR #47 OR #48 OR #49 OR #50 OR #51 OR #52 OR #53 OR #54 OR #55 OR #56 OR #57 OR #58 OR #59 OR #60 OR #61 OR #62 OR #63 OR #64 OR #65 OR #66 OR #67 OR #68 OR #69 OR #70 OR #71 OR #72 OR #73 or #74 |
| 76 | app-based.tw,kf. | app-based.tw,kf. | TI usage OR AB usage | Digital Divide/ |
| 77 | 40 or 41 or 42 or 43 or 44 or 45 or 46 or 47 or 48 or 49 or 50 or 51 or 52 or 53 or 54 or 55 or 56 or 57 or 58 or 59 or 60 or 61 or 62 or 63 or 64 or 65 or 66 or 67 or 68 or 69 or 70 or 71 or 72 or 73 or 74 or 75 | 39 or 40 or 41 or 42 or 43 or 44 or 45 or 46 or 47 or 48 or 49 or 50 or 51 or 52 or 53 or 54 or 55 or 56 or 57 or 58 or 59 or 60 or 61 or 62 or 63 or 64 or 65 or 66 or 67 or 68 or 69 or 70 or 71 or 72 or 73 or 74 or 75 or 76 | TI use N2 smart#phone OR AB use N2 smart#phone | Internet Access/ |
| 78 | Digital divide/ | internet access/ | TI use N2 cell#phone OR AB use N2 cell#phone | Internet Use/ |
| 79 | Internet access/ or “Internet use”/ | digital divide/ | TI use N2 computer OR AB use N2 computer | Exp Health Services Accessibility/ |
| 80 | health services accessibility/ or access to primary care/ or health equity/ or right to health/ or universal health care/ | health care access/ or access to medication/ or access to treatment/ or health care availability/ or primary care access/ | TI use N2 mobile* OR AB use N2 mobile* | Delivery of Healthcare/ |
| 81 | “Delivery of healthcare”/ and digital health/ and telemedicine/ | “digi* divi*”.tw,kf. | TI use N2 tech* OR AB use N2 tech* | Cell Phone Use/ |
| 82 | “digi* divi*”.tw,kf. | access*.tw,kf. | TI use N2 app* OR AB use N2 app* | Internet Use/ |
| 83 | access*.tw,kf. | avail*.tw,kf. | TI use N2 internet OR AB use N2 internet | (digi* NEXT divi*):ti,ab,kw |
| 84 | avail*.tw,kf. | usage.tw,kf. | S69 OR S70 OR S71 OR S72 OR S73 OR S74 OR S75 OR S76 OR S77 OR S78 OR S79 OR S80 OR S81 OR S82 OR S83 | (access*):ti,ab,kw |
| 85 | usage.tw,kf. | "use" adj2 ("smart*" "phone*").tw,kf. | (MH "Attitude") OR (MH "Attitude to Computers") OR (MH "Attitude to Medical Treatment+") OR (MH "Attitude to Health+") OR (MH "Attitude to Illness") OR (MH "Patient Attitudes") | (avail*):ti,ab,kw |
| 86 | "use" adj2 ("smart*" "phone*").tw,kf. | "use" adj2 ("cell*" "phone*").tw,kf. | TI attitude* OR AB attitude* | (usage):ti,ab,kw |
| 87 | "use" adj2 ("cell*" "phone*").tw,kf. | "use" adj2 ("cell*" "phone*").tw,kf. | TI *engag* OR AB *engag* | (use near/3 smart*phone*):ti,ab,kw |
| 88 | "use" adj2 "computer*".tw,kf. | "use" adj2 "computer*".tw,kf. | TI thought OR AB thought | (use near/3 cell*phone*):ti,ab,kw |
| 89 | "use" adj2 "mobile*".tw,kf. | "use" adj2 "mobile*".tw,kf. | TI belief OR AB belief | (use near/3 computer*):ti,ab,kw |
| 90 | "use" adj2 "internet*".tw,kf. | "use" adj2 "internet*".tw,kf. | TI view OR AB view | (use near/3 mobile*):ti,ab,kw |
| 91 | "use" adj2 "tech*".tw,kf. | "use" adj2 "tech*".tw,kf. | TI opinion OR AB opinion | (use near/3 internet*):ti,ab,kw |
| 92 | "use" adj2 "app*".tw,kf. | "use" adj2 "app*".tw,kf. | S85 OR S86 OR S87 OR S88 OR S89 OR S90 OR S91 | (use near/3 tech*):ti,ab,kw |
| 93 | 78 or 79 or 80 or 81 or 82 or 83 or 84 or 85 or 86 or 87 or 88 or 89 or 90 or 91 or 92 | 78 or 79 or 80 or 81 or 82 or 83 or 84 or 85 or 86 or 87 or 88 or 89 or 90 or 91 or 92 | TI barrier OR AB barrier | (use near/3 app*):ti,ab,kw |
| 94 | attitude/ or attitude to computers/ or attitude to health/ or health knowledge, attitudes, practice/ or “treatment adherence and compliance”/ or “patient acceptance of health care”/ or patient satisfaction/ or treatment refusal/ | attitude/ or attitude to computers/ or attitude to disability/ or attitude to health/ or attitude to illness/ or health personnel attitude/ or patient attitude/ | TI facilitat* OR AB facilitat* | #76 OR #77 OR #78 OR #79 OR #80 OR #81 OR #82 OR #83 OR #84 OR #85 OR #86 OR #87 OR #88 OR #89 OR #90 OR #91 OR #92 OR #93 |
| 95 | “attitude*”.tw,kf. | “attitude*”.tw,kf. | TI motivat* OR AB motivat* | MeSH descriptor: [Attitude] this term only |
| 96 | “*engag*”.tw,kf. | “*engag*”.tw,kf. | TI enabl* OR AB enabl* | MeSH descriptor: [Attitude to Computers] this term only |
| 97 | “thought*”.tw,kf. | “thought*”.tw,kf. | TI challeng* OR AB challeng* | MeSH descriptor: [Attitude to Health] explode all trees |
| 98 | “belief*”.tw,kf. | “belief*”.tw,kf. | TI "factors influencing" OR AB "factors influencing" | (attitude*):ti,ab,kw |
| 99 | “view*”.tw,kf. | “view*”.tw,kf. | S93 OR S94 OR S95 OR S96 OR S97 OR S98 | (*engag*):ti,ab,kw |
| 100 | “opinion*”.tw,kf. | “opinion*”.tw,kf. | **S1: Older adults + perioperative + digital health literacy**  S10 AND S19 AND S33 | (thought*):ti,ab,kw |
| 101 | 94 or 95 or 96 or 97 or 98 or 99 or 100 | 94 or 95 or 96 or 97 or 98 or 99 or 100 | **S2: Older adults + perioperative + digital access and use + tools**  S10 AND S19 AND S68 AND S84 | (belief*):ti,ab,kw |
| 102 | barrier*.tw,kf | communication barrier/ | **S3: Older adults + perioperative + attitudes + digital tools**  S10 AND S19 AND S68 AND S92 | (view*):ti,ab,kw |
| 103 | facilitat*.tw,kf. | barrier*.tw,kf | **S4: Older adults + perioperative + barriers/facilitators + digital tools**  S10 AND S19 AND S68 AND S99 | (opinion*):ti,ab,kw |
| 104 | motivat*.tw,kf. | motivat*.tw,kf. | **S5: Final search**  S100 OR S101 OR S102 OR S103 | #95 OR #96 OR #97 OR #98 OR #99 OR #100 OR #101 OR #102 OR #103 |
| 105 | enabl*.tw,kf. | enabl*.tw,kf. |  | MeSH descriptor: [Precipitating Factors] explode all trees |
| 106 | challeng*.tw,kf. | facilitat*.tw,kf. |  | (barrier*):ti,ab,kw |
| 107 | “Factors influencing”.tw,kf. | challeng*.tw,kf. |  | (facilitator*):ti,ab,kw |
| 108 | 102 or 103 or 104 or 105 or 106 or 107 | “Factors influencing”.tw,kf. |  | (motivat*):ti,ab,kw |
| 109 | **S1: Older adults + perioperative + digital health literacy**  9 and 23 and 39 | 102 or 103 or 104 or 105 or 106 or 107 or 108 |  | (enabl*):ti,ab,kw |
| 110 | **S2: Older adults + perioperative + digital access and use + digital tools**  9 and 23 and 77 and 93 | **S1: Older adults + perioperative + digital health literacy**  8 and 21 and 38 |  | (challeng*):ti,ab,kw |
| 111 | **S3: Older adults + perioperative + attitudes + digital tools**  9 and 23 and 77 and 101 | **S2: Older adults + perioperative + digital access and use + digital tools**  8 and 21 and 77 and 93 |  | (“factors influencing”):ti,ab,kw |
| 112 | **S4: Older adults + perioperative + barriers/facilitators + digital tools**  9 and 23 and 77 and 109 | **S3: Older adults + perioperative + attitudes + digital tools**  8 and 21 and 77 and 101 |  | #103 OR #104 OR #105 OR #106 OR #107 OR #108 OR #109 |
| 113 | **S5: Final search**  109 OR 110 OR 111 OR 112 | **S4: Older adults + perioperative + barriers/facilitators + digital tools**  8 and 21 and 77 and 109 |  | **S1: Older adults + perioperative + digital health literacy**  #10 AND #19 AND #33 |
| 114 |  | **S5: Final search**  110 OR 111 OR 112 OR 113 |  | **S2: Older adults + perioperative + digital access and use + digital tools**  #10 AND #19 AND #68 AND #84 |
| 115 |  |  |  | **S3: Older adults + perioperative + attitudes + digital tools**  #10 AND #19 AND #68 AND #92 |
| 116 |  |  |  | **S4: Older adults + perioperative + barriers/facilitators + digital tools**  #10 AND #19 AND #68 AND #99 |
| 117 |  |  |  | **S5: Final search**  #113 OR #114 OR #115 OR #116 |
